# Research on Vibration Signal Processing and Fault Diagnosis Algorithms for High-Pressure Quintuplex Pumps

**DOI:** 10.3390/s26154917

**Published:** 2026-08-04

**Authors:** Zewei Liu, Yuanchen Zhu, Zhigang Zhang, Tao Zhang

**Affiliations:** School of Mechanical Engineering, Sichuan University, Chengdu 610065, China; liuzewei@stu.scu.edu.cn (Z.L.); zhuyuanchen@stu.scu.edu.cn (Y.Z.); nic6700@scu.edu.cn (T.Z.)

**Keywords:** quintuplex pumps, fault diagnosis, blind source separation, angular-domain resampling, Gramian Angular Field, recurrence plot, unsupervised cross-condition diagnosis, vision transformer

## Abstract

High-pressure quintuplex pumps are key components in oil and gas drilling systems, but their fault diagnosis remains challenging due to severe inter-cylinder vibration crosstalk, operating-condition variability, and limited labeled samples. To address these issues, this paper proposes an integrated vibration-based diagnostic framework, termed VICA-DA-ViT. First, a VMD–FastICA-based blind source separation strategy is used to suppress inter-cylinder crosstalk and extract more discriminative vibration components from mixed signals. Then, angular-domain resampling and normalization are applied to reduce speed-induced non-stationarity and improve cycle consistency. The processed one-dimensional signals are further converted into two-dimensional GAF–RP representations, where GAF captures global temporal correlations and RP provides complementary recurrence information. Finally, a ViT-based domain-adaptive diagnostic framework is constructed for unsupervised cross-condition diagnosis by integrating domain-adversarial pretraining, weighted bidirectional sample pairing, and Gaussian-mixture-based confidence filtering around a standard Vision Transformer backbone. Rather than modifying the internal architecture of ViT, the proposed framework improves cross-condition generalization by combining signal decoupling, angular-domain alignment, and domain-adaptation strategies for high-pressure quintuplex pump fault diagnosis. Experiments on a quintuplex pump dataset under multiple operating conditions show that the proposed framework achieves an average cross-condition accuracy of 87.42%, outperforming CD-Trans by 11.94%. These results demonstrate that VICA-DA-ViT can effectively improve the robustness of small-sample cross-condition fault diagnosis for high-pressure quintuplex pumps.

## 1. Introduction

High-pressure quintuplex pumps are indispensable in oil and gas drilling and transportation systems, as they provide the critical circulation flow required for drilling-fluid loops [1]. However, prolonged erosion and cyclic hydraulic shocks can induce failures in critical fluid-end components such as valves, potentially leading to unplanned downtime and safety hazards [2]. Consequently, accurate and reliable fault diagnosis of high-pressure quintuplex pumps is essential to ensuring stable and safe field operation.

Currently, vibration signal-based fault detection is the predominant method, as it enables non-intrusive, continuous monitoring and exhibits high sensitivity to early-stage mechanical anomalies [3]. In practical vibration-based diagnosis, signal processing is commonly performed to enhance weak fault characteristics, including band-pass filtering, wavelet-threshold denoising, envelope analysis, and spectral-kurtosis-based impulsiveness enhancement, as well as time–frequency transforms such as STFT/CWT and generalized S-transform representations. These preprocessing and representation techniques have been widely combined with machine learning or deep learning classifiers to improve fault recognition under noisy environments [4]. However, for high-pressure quintuplex pumps, strong structural coupling and hydraulic excitations often introduce pronounced inter-cylinder crosstalk, making the measured vibration a mixture of responses from multiple cylinders, which cannot be fundamentally disentangled by conventional denoising or time–frequency enhancement alone. Therefore, blind source separation (BSS) methods, especially ICA-based approaches, are introduced to recover cylinder-related components from mixed observations [5]; in single-sensor settings, decomposition-assisted BSS (e.g., Variational Mode Decomposition (VMD) followed by FastICA) further provides an effective way to suppress crosstalk before subsequent diagnosis.

Wang et al. proposed an EEMD–ICA method to recover compound-fault information from single-channel measurements, though EEMD remains limited by residual noise and mode mixing [6]. Sun and Fang introduced a VMD–KICA framework, demonstrating that VMD provides superior resistance to modal aliasing as a front-end decomposition tool [7]. Yang and Li developed an integrated framework combining improved GOOSE-VMD, RobustICA, and CYCBD, demonstrating the efficacy of robust decomposition for complex mechanical signals [8]. More recently, Wang et al. implemented a VMD-based virtual-channel BSS method using Tukey M estimation, proving that precise spectral division further enhances fault source separation accuracy [9].

Despite the effectiveness of VMD, relying solely on raw decomposed components or their derived statistical features often fails to provide sufficient discriminative information for high-accuracy classification. This limitation stems from the fact that these components primarily offer a frequency-domain perspective, which is challenging for diagnostic models to interpret when dealing with complex, non-stationary patterns. To bridge this gap, the Gramian Angular Field (GAF) is employed to transform one-dimensional time series into a high-dimensional feature space. By encoding the temporal dependencies and amplitude variations into two-dimensional images, GAF preserves the integrity of the signal’s dynamic characteristics while providing a more intuitive topological representation for feature extraction.

Wang and Oates first proposed the Gramian Angular Field (GAF) framework, leveraging computer-vision techniques to achieve efficient time-series classification [10]. Kim et al. subsequently enhanced GAF’s structured input capabilities through patch-level contrastive learning [11]. In the field of pump fault diagnosis, Li et al. validated the advantages of GAF-based image fusion for preserving rich structural information under multiple operating conditions [12], while Tang et al. and Shen et al. demonstrated that GAF can reconstruct robust high-dimensional features, thereby improving the extraction performance of deep learning models [13,14].

Deep learning techniques have been widely adopted in the field of mechanical equipment fault diagnosis [15]. Among them, convolutional neural networks (CNNs) enable end-to-end feature learning from raw vibration sequences or their time–frequency representations, reducing reliance on handcrafted indicators [16]. To further emphasize fault-relevant information and suppress interference, attention modules (e.g., channel/spatial attention) are often integrated with CNN backbones to adaptively reweight features [17]. Beyond CNNs, recurrent architectures such as LSTM/GRU have also been employed to model temporal dependencies in vibration signals and improve robustness under noise [18]. When labeled fault samples are scarce, unsupervised representation learning with autoencoders (e.g., variational/denoising autoencoders) [19,20] can leverage abundant unlabeled data to learn compact and noise-tolerant latent features, while self-supervised contrastive learning pre-training has shown strong label efficiency for machinery diagnosis [21]. Nevertheless, practical industrial data are frequently collected under varying operating conditions (e.g., load, speed, and sensor placement), leading to domain shifts that degrade diagnostic performance; deep domain adaptation and adversarial training aim to learn domain-invariant representations to enhance generalization [22]. Conventional unsupervised domain adaptation strategies, including class-balanced self-training [23], source-data-absent transfer schemes [24], self-supervised learning under very limited labels [25], and optimal transport-based adaptation [26], have also provided important perspectives for improving cross-condition generalization in fault diagnosis. More recently, transformer-based architectures built upon self-attention have been introduced to capture long-range dependencies globally, and Vision Transformers (ViT)-style models and their time-series variants have demonstrated promising results for vibration-based and cross-domain fault diagnosis.

Hassija et al. reported that ViT outperforms conventional CNNs on multiple recognition benchmarks [27]. Dosovitskiy et al. proposed Vision Transformer (ViT), which directly treats an image as a sequence of patches and learns global relationships through self-attention [28]. In machinery diagnosis, Chu et al. proposed a cross-domain fault diagnosis method fusing acoustic and vibration signals by vision transformer [29], while Zhang et al. developed TSViT for rotating machinery fault diagnosis and showed the potential of transform-er-based visual modeling for complex fault pattern recognition [30].

Most existing studies on high-pressure reciprocating pumps focus on either learning-based cross-condition adaptation or signal processing in isolation. Few works jointly consider severe inter-cylinder crosstalk suppression, cycle-aligned preprocessing under speed fluctuations, and robust cross-condition diagnosis under limited labels in a unified framework. Motivated by these challenges, an integrated workflow, named VICA-DA-ViT, for high-pressure quintuplex pump vibration processing and unsupervised cross-condition fault diagnosis is proposed in this paper, consisting of crosstalk suppression, angular-domain alignment, and domain-invariant representation learning.

The main contributions of this study are as follows:

1. A customized VMD–FastICA blind source separation scheme is proposed to decouple strong inter-cylinder interference. By strategically reorganizing informative modes and auxiliary channels, this scheme effectively expands the observation space, enabling the precise extraction of cylinder-specific vibration components from single-sensor data.
2. A GAF-based one-dimensional-to-two-dimensional feature encoding strategy is proposed for quintuplex pump vibration signals. By transforming preprocessed one-dimensional signals into structured two-dimensional representations through Gramian Angular Field encoding, the proposed method preserves temporal correlations and structural fault information more effectively, thereby providing more informative inputs for subsequent visual diagnosis.
3. A ViT-based domain-adaptive diagnostic framework is developed for unsupervised cross-condition diagnosis. The framework retains the standard Vision Transformer backbone and improves cross-condition transferability by integrating domain-adversarial pretraining, GMM-guided confidence filtering, and weighted bidirectional sample pairing. Therefore, the novelty lies in the coordinated diagnostic framework and domain-adaptation strategy rather than in architectural modification of the ViT backbone.

The remainder of this paper is organized as follows. Section 2 details the proposed VICA-DA-ViT methodology, including the VMD–FastICA crosstalk suppression scheme, angular-domain resampling, and the ViT-based domain-adaptive diagnostic framework. Section 3 presents the experimental setup, performance analysis of the diagnostic pipeline, and comparative results under multiple operating conditions. Section 4 concludes the paper and discusses future work.

## 2. Method

To address the core challenges in high-pressure quintuplex pump diagnosis—specifically the severe inter-cylinder crosstalk from structural coupling, non-stationarity driven by speed fluctuations, and poor model generalization under small-sample cross-condition scenarios—this paper proposes an integrated unsupervised diagnostic framework. The methodology transitions from VICA-based crosstalk suppression and angular-domain resampling to feature reconstruction and domain-invariant representation learning via a ViT-based domain-adaptive diagnostic framework. Specifically, the VMD-FastICA (VICA) scheme is tailored to decouple single-channel signals by constructing a virtual observation space from informative modes. Subsequently, angular-domain resampling eliminates speed-induced smear by aligning cycles in the rotation domain. Finally, the decoupled signals are transformed into multi-channel GAF-RP images and fed into the proposed VICA-DA-ViT framework, which incorporates GMM-based confidence filtering and domain-adversarial pretraining to achieve robust, label-efficient diagnosis across varying operating conditions.

### 2.1. Signal Decoupling via VMD-FastICA with Virtual Channel Expansion

The technical objective of the VICA scheme is to resolve the underdetermined blind source separation (BSS) problem inherent in the single-sensor monitoring of quintuplex pumps. The vibration signals of these high-pressure pumps are characterized by intensive non-stationarity and complex spectral overlaps, where high-energy hydraulic excitations frequently submerge the subtle impulsive signatures of mechanical faults. Due to the compact multi-cylinder structure, the measured signal is not a pure representation of a single source but a convoluted mixture of inter-cylinder crosstalk. To fundamentally decouple these components, the proposed methodology follows a logical transition from adaptive signal partitioning to statistical decoupling. Specifically, the raw vibration is first decomposed into distinct frequency-limited subspaces using Variational Mode Decomposition (VMD), followed by a dual-criterion mode selection to construct a virtual observation space. Finally, the inter-cylinder crosstalk is suppressed via Fast Independent Component Analysis (FastICA), transforming the complex structural mixture into independent sources to ensure that interference is suppressed at the physical source level.

**Figure 1:**
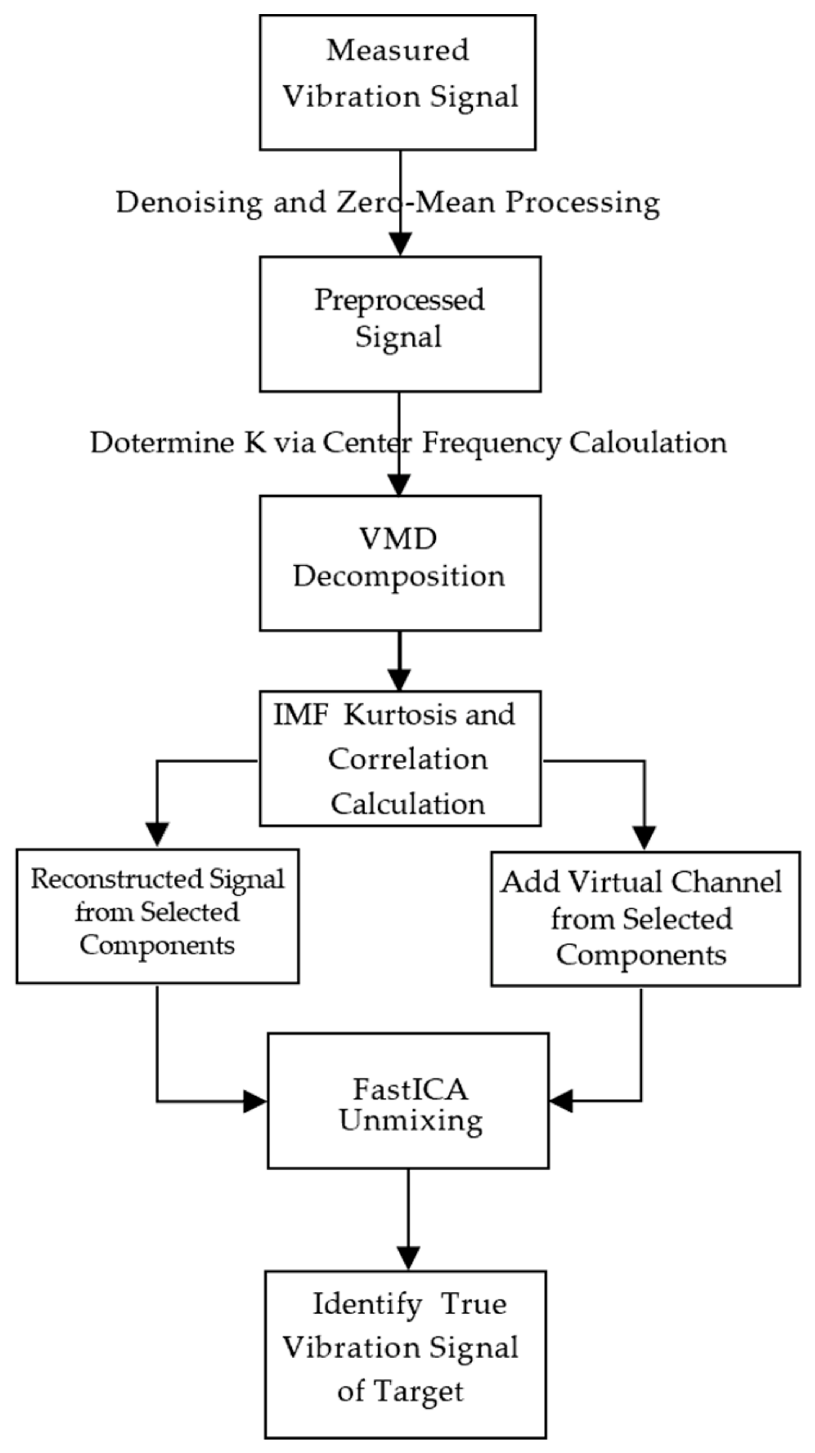
Flowchart of the proposed VMD–FastICA-based signal processing procedure.

The implementation of this hierarchical decoupling strategy is visualized in Figure 1, which illustrates the systematic integration of VMD and FastICA. The detailed operational procedure is executed through the following stages in Table 1.

Variational Mode Decomposition (VMD) formulates signal decomposition as a constrained variational optimization problem. Given a pump vibration signal s(t), VMD models it as a superposition of K intrinsic mode functions (IMFs), *v_k_*(*t*) (*k* = 1, …, K), where *v_k_*(*t*) denotes the *k*-th mode and *ω_k_* is its center frequency. The objective of VMD is to estimate *v_k_(t)* and *ω_k_* by minimizing the sum of the modes’ estimated bandwidths under a reconstruction constraint. The specific implementation steps of the VMD algorithm are summarized as follows.

(1) For each IMF mode *v_k_*(*t*), the Hilbert transform is applied to obtain its analytic signal, as shown in Equation (1).

(1) $$\left[\delta \left(t\right)+\frac{j}{\pi t}\right]*v_{k}\left(t\right)$$

(2) The analytic signal of each mode is then demodulated to baseband using the current estimate of its center frequency *ω_k_*, yielding a frequency-shifted representation centered at zero frequency, as shown in Equation (2).

(2) $$\left\{\left[\delta \left(t\right)+\frac{j}{\pi t}\right]*v_{k}\left(t\right)\right\}e^{-j\omega_{k}t}$$

(3) The bandwidth of each IMF mode is estimated by computing the squared L2 norm of the temporal gradient of the demodulated signal, as indicated in Equation (3).

(3) $$\left\{\begin{array}{l} \min_{\left\{v_{k}\},\{a_{k}\right\}}\{\sum_{k}\|\sigma_{i}[(\delta (t)+\frac{j}{\pi t})*v_{k}(t)]e^{-j\omega_{k}t}{\|}_{2}^{2}\}\\ s.t.\sum_{k}v_{k}=s(t) \end{array}\right.$$

where $\left\{vₖ\}\;=\;\{v_{1},\;v_{2},\;\ldots ,\;v_{K}\right\}$ denotes the *K* IMFs obtained by VMD, and $\left\{\omega ₖ\}\;=\;\{\omega_{1},\;\omega_{2},\;\ldots ,\;\omega_{K}\right\}$ denotes their corresponding center frequencies. The symbol * denotes convolution, *δ*(*t*) is the Dirac delta function, ands(t) represents the measured vibration signal.

(4) By introducing a quadratic penalty factor *α* and a Lagrange multiplier *λ*(*t*), the constrained problem can be written in the augmented Lagrange form as:

(4) $$L\left(\left\{v_{k}\right\},\left\{\omega_{k}\right\},\lambda \right)=\alpha \sum_{k}\left\{{\left\|\partial_{t}\left[\left(\delta (t)+\frac{j}{\pi t}\right)*v_{k}(t)\right]e^{-j\omega_{k}t}\right\|}_{2}^{2}+{\left\|s(t)-\sum_{k}v_{k}(t)\right\|}_{2}^{2}+\langle \lambda (t),s(t)-\sum_{k}v_{k}(t)\rangle \right\}$$

(5) The augmented Lagrange is solved using the Alternating Direction Method of Multipliers (ADMM), which alternately updates the modes and the dual variable. The update of *v_k_* in the frequency domain is given in Equation (5):

(5) $${\hat{v}}_{k}^{n+1}(\omega )=\frac{\hat{s}(\omega )-\sum_{i<k}{\hat{v}}_{i}^{n+1}(\omega )-\sum_{i>k}{\hat{v}}_{i}^{n}(\omega )+{\hat{\lambda }}^{n}(\omega )/2}{1+2\alpha {\left(\omega -\omega_{k}^{n}\right)}^{2}}$$

where ω denotes the frequency variable. Quantities with a hat(^) denote the Fourier transforms of the corresponding time-domain signals; for example, ${\hat{v}}_{k}^{n}(\omega )$, *ŝ*(*ω*), and *λ^n^(ω)* correspond to $v_{k}^{n}(t)$, *s*(*t*), and *λ*(*t*), respectively.

(6) The center frequency *ω_k_* and the Lagrange multiplier *λ* are updated according to Equations (6) and (7), where *γ* is the dual ascent step size.

(6) $$\omega_{k}^{n+1}=\frac{\int_{0}^{\infty }\omega {\left|{\hat{v}}_{k}^{n+1}(\omega )\right|}^{2}d\omega }{\int_{0}^{\infty }{\left|{\hat{v}}_{k}^{n+1}(\omega )\right|}^{2}d\omega }$$

(7) $${\hat{\lambda }}^{n+1}(\omega )={\hat{\lambda }}^{n}(\omega )+\gamma \left[\hat{s}(\omega )-\sum_{k=1}^{K}{\hat{v}}_{k}^{n+1}(\omega )\right]$$

(7) The VMD algorithm iteratively updates the above variables until the convergence criterion in Equation (8) is satisfied:

(8) $$\sum_{k=1}^{K}\left(\frac{{\left\|{\hat{v}}_{k}^{n+1}-{\hat{v}}_{k}^{n}\right\|}_{2}^{2}}{{\left\|{\hat{v}}_{k}^{n}\right\|}_{2}^{2}}\right)<\varepsilon$$

The overall VMD procedure is summarized above. The iterations terminate when the relative change between two consecutive updates falls below a predefined tolerance ε.

The kurtosis criterion is a fourth-order statistical measure that characterizes the impulsiveness of a signal. It is defined as the normalized fourth-order central moment:

(9) $$K=\frac{E{\left(x-\mu \right)}^{4}}{\sigma^{4}}$$

where μ is the mean, σ is the standard deviation, and E(·) denotes the expectation operator. A higher kurtosis generally indicates more significant impulsive components. In quintuplex pump fault diagnosis, periodic fault-induced impacts tend to increase the kurtosis of informative IMFs, whereas noise-dominated components typically exhibit kurtosis values close to 3 (Gaussian-like).

The Pearson correlation coefficient is used to quantify the linear similarity between two signals. By computing the correlation between the preprocessed signal and each IMF, IMFs with higher correlation are selected for signal reconstruction, thereby retaining fault-related information while suppressing irrelevant components. The correlation coefficient is calculated as:

(10) $$\rho_{XY}=\frac{Cov\left(X,Y\right)}{\sqrt{D\left(X\right)\sqrt{D\left(Y\right)}}}$$

where X denotes the preprocessed signal, and Y denotes the IMF component.

Fast Independent Component Analysis (FastICA) is a computationally efficient independent component analysis (ICA) algorithm widely used for blind source separation. Given multichannel observations that are assumed to be linear mixtures of statistically independent sources, FastICA estimates a demixing matrix by maximizing non-Gaussianity, which is commonly measured using negentropy. Compared with conventional ICA methods, FastICA adopts a fixed-point iteration scheme, typically leading to faster convergence and reduced computational cost. Therefore, FastICA is suitable for separating coupled vibration components and improving the reliability of subsequent feature extraction.

The objective of FastICA is to recover statistically independent source signals from multichannel vibration measurements. Specifically, it aims to separate M independent sources *S*(*t*) from N observed vibration signals *X*(*t*) under a linear mixing assumption:

(11) $$X\left(t\right)=A\left(t\right)*S\left(t\right)+V\left(t\right)$$

where *X*(*t*) is the N-dimensional observation vector, *S*(*t*) is the M-dimensional unknown source vector, *A*(*t*) is the mixing matrix, and *V*(*t*) denotes additive noise.

### 2.2. Two-Dimensional Feature Encoding Based on Multi-Channel GAF–RP

After crosstalk suppression via VMD-FastICA, angular-domain resampling and normalization, the vibration signals of the high-pressure quintuplex pump remain one-dimensional time-series data that only reflect the amplitude variation over time and cannot be directly input into the ViT-based domain-adaptive diagnostic model tailored for two-dimensional images. Given that the multi-head self-attention mechanism of ViT excels at capturing the global correlations and structural features of two-dimensional data, the Gramian Angular Field (GAF) is introduced to realize 1D-to-2D transformation. Specifically, the preprocessed one-dimensional signals are first normalized to the range of [−1,1] and then mapped to the polar coordinate system to generate the GASF (encoding amplitude correlations) and GADF (capturing temporal variations). In addition, the Recurrence Plot (RP) is incorporated as the third channel to enhance the signal similarity features. These three components are stacked to form a 224 × 224 × 3 pseudo-RGB two-dimensional image, which not only fills the adaptation gap between one-dimensional data and two-dimensional models but also completely preserves the fault temporal information in the signals and enhances feature discriminability. This provides high-quality two-dimensional feature support for the subsequent cross-domain fault feature learning of the ViT-based domain-adaptive diagnostic model.

To satisfy the image-like input requirement of the Vision Transformer (ViT), each one-dimensional vibration sample is transformed into a two-dimensional representation by encoding pairwise relationships among signal points. Given a 1D vibration signal $x=[x_{1},x_{2},\ldots ,x_{L}]\in R^{L}$, resampling is first performed to convert the signal to a fixed length N, so that a consistent image resolution is obtained. In this work, Nis set to 224:

(12) $$x\in {\mathbb{R}}^{L}\Rightarrow \bar{x}\in {\mathbb{R}}^{N},\;N=224$$

Then, $\bar{x}$ is normalized into [−1,1] as

(13) $${\tilde{x}}_{i}=2\cdot \frac{{\bar{x}}_{i}-\min(\bar{x})}{\max(\bar{x})-\min(\bar{x})+\varepsilon }-1,\;{\tilde{x}}_{i}\in [-1,1]$$

where ε is a small constant to avoid numerical instability. Next, each normalized sample is mapped into an angular space:

(14) $$\phi_{i}=\arccos\left({\tilde{x}}_{i}\right)$$

Based on the Gramian Angular Field, two complementary 2D maps are constructed to characterize the global dependencies of the signal:

(15) $$GASF_{i,j}=\cos\left(\phi_{i}+\phi_{j}\right),\;i,j\in \{1,\ldots ,N\}$$

(16) $$GADF_{i,j}=\sin\left(\phi_{i}-\phi_{j}\right),\;i,j\in \{1,\ldots ,N\}$$

In addition, a recurrence-style similarity map is introduced as the third channel to form a pseudo-RGB input:

(17) $$RP_{i,j}=\exp\left(-\frac{\left|{\tilde{x}}_{i}-{\tilde{x}}_{j}\right|}{\sigma +\varepsilon }\right),\;\sigma =mean_{i,j}\left(\left|{\tilde{x}}_{i}-{\tilde{x}}_{j}\right|\right)$$

Finally, the three maps are stacked along the channel dimension to obtain an image-like representation:

(18) $$I=Stack(GASF,GADF,RP)\in R^{N\times N\times 3}=R^{224\times 224\times 3}$$

GAF is adopted to transform 1D vibration signals into a 2D representation, as the two-dimensional mapping explicitly encodes global dependencies through pairwise relationships: each pixel $\left(i,j\right)$ is deterministically computed from the i-th and j-th samples, producing structured textures that better capture repetitive fault patterns and long-range correlations. In contrast, directly processing 1D sequences typically relies more on local operations or limited receptive fields, which may be insufficient to model such global relations. Moreover, mapping signals to a fixed-size 2D space reduces sensitivity to length variations and partially alleviates the influence of raw amplitude discrepancies, encouraging the model to focus on more stable structural information. This improves robustness to noise and operating-condition shifts, while providing an image-like input that is naturally compatible with ViT patch tokenization and global feature modeling.

### 2.3. ViT-Based Domain-Adaptive Diagnostic Framework for Cross-Condition Diagnosis

The proposed ViT-based domain-adaptive diagnostic framework is designed to address small-sample learning, domain shift, and complex temporal dependence in cross-condition fault diagnosis of high-pressure quintuplex pumps. In this framework, the standard Vision Transformer is retained as the backbone for pseudo-RGB vibration-image feature extraction. The enhancement of diagnostic performance is achieved not by modifying the internal architecture of ViT, but by introducing domain-adaptation strategies around the ViT backbone, including domain-adversarial pretraining, weighted bidirectional source–target sample pairing, and GMM-based confidence filtering. These strategies encourage the model to learn domain-invariant and fault-discriminative representations under varying operating conditions.

It first converts the 1D vibration signals (after VMD-FastICA crosstalk suppression, angular-domain resampling and normalization) into 2D feature maps via GAF, where GASF encodes global amplitude correlations, GADF captures temporal variation trends, and RP is introduced to characterize inter-signal-point similarity; the three are stacked into a 224 × 224 × 3 pseudo-RGB image, which meets ViT’s input requirements while preserving the fault temporal dependence and global correlation information of vibration signals. The encoder layer takes ViT’s classic Patch embedding-MSA-MLP as the basic module: it optimizes Patch embedding and positional encoding with domain-adaptive fine-tuning to reduce cross-condition positional offset interference, and integrates domain-adversarial pretraining and bidirectional weighted sample pairing modules to force the model to learn working condition-independent fault features and screen high-consistency source-target sample pairs, thus alleviating feature confusion caused by domain shift.

For the scarcity of labeled data in the target domain, the proposed VICA-DA-ViT framework adopts a dual-loss joint training strategy of GMM-based pseudo-label supervision and cluster consistency constraint: it generates soft pseudo-labels for target domain samples and filters low-quality ones with a 0.5 confidence threshold, then designs a total loss function fusing confidence-weighted cross-entropy loss and cluster consistency loss to enhance feature compactness and inter-class discriminability, finally mapping the class token features to the probability distribution of four fault types (normal condition, inlet-valve fault, discharge-valve fault, dual-valve fault) via a linear classifier. In addition, the proposed framework features excellent small-sample robustness (diagnostic accuracy improved by over 12% compared with traditional ViT) and industrial-level computational efficiency (single-sample inference time within 10 ms), well adapting to the actual demands of on-site fault diagnosis of quintuplex pumps.

**Figure 2:**
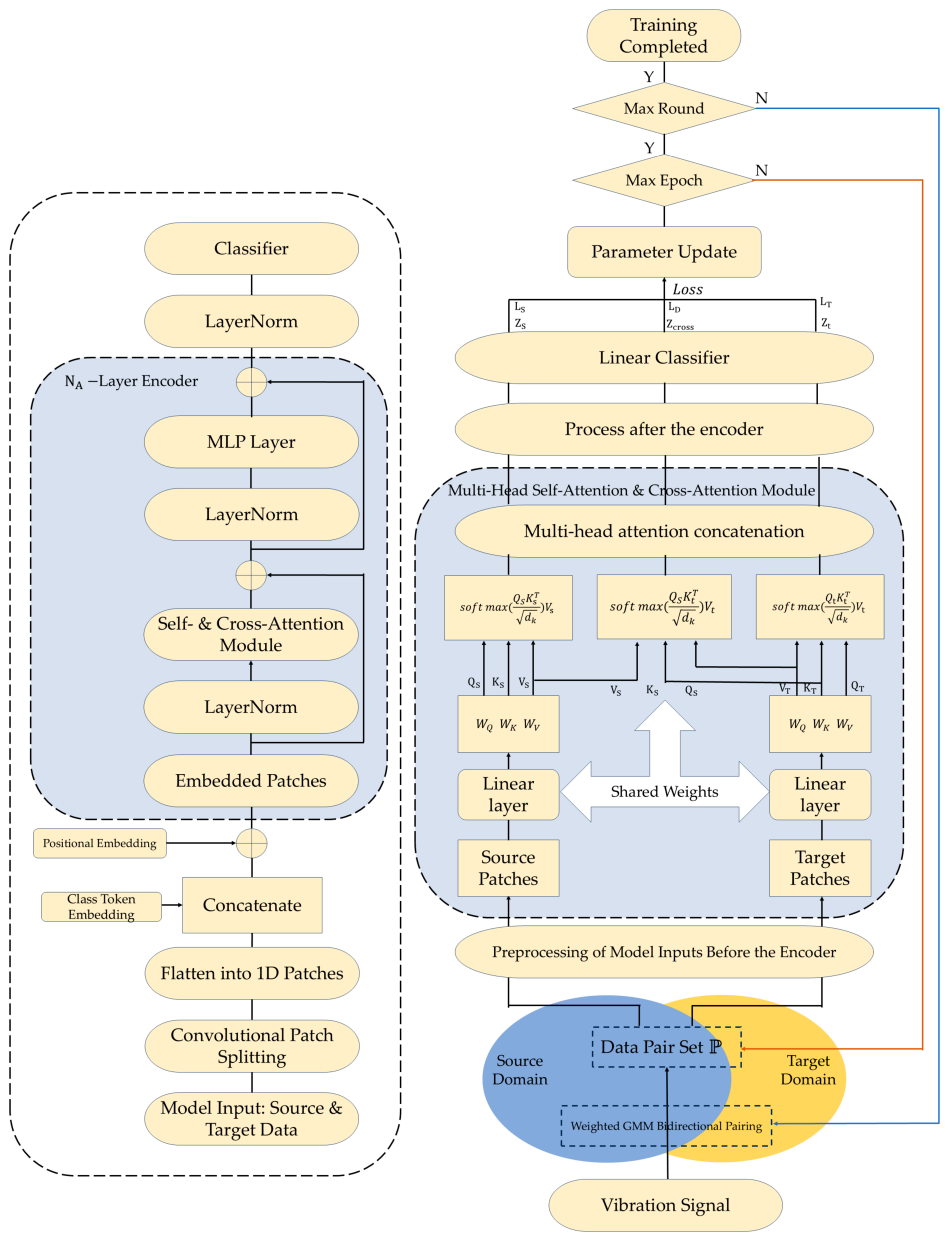
Overall structure of the ViT-based domain-adaptive diagnostic framework.

The integration of these strategies and the overall information flow of the ViT-based domain-adaptive diagnostic framework are visually summarized in Figure 2, which highlights the transition from raw feature extraction to refined fault classification.

The proposed VICA-DA-ViT framework operates through a coordinated data flow designed to achieve domain-invariant feature mapping. The process begins at the Input Layer, where source and target domain signals are coupled via a Weighted GMM Bidirectional Pairing module, ensuring that the model captures shared underlying fault physics despite condition variations. These paired inputs are then processed by the NA-Layer Encoder, which serves as the core feature extractor. Within this encoder, the Self- & Cross-Attention Module leverages shared weights to project multi-domain data into a unified latent space, effectively suppressing condition-specific interference. Finally, the diagnostic performance is refined through a Tripartite Loss Function, which integrates supervised source labels with GMM-generated pseudo-labels for the target domain. This iterative parameter update loop, guided by a 0.5 confidence threshold, ensures that the model converges to a state characterized by both high inter-class discriminability and robust cross-condition generalization.

**Figure 3:**
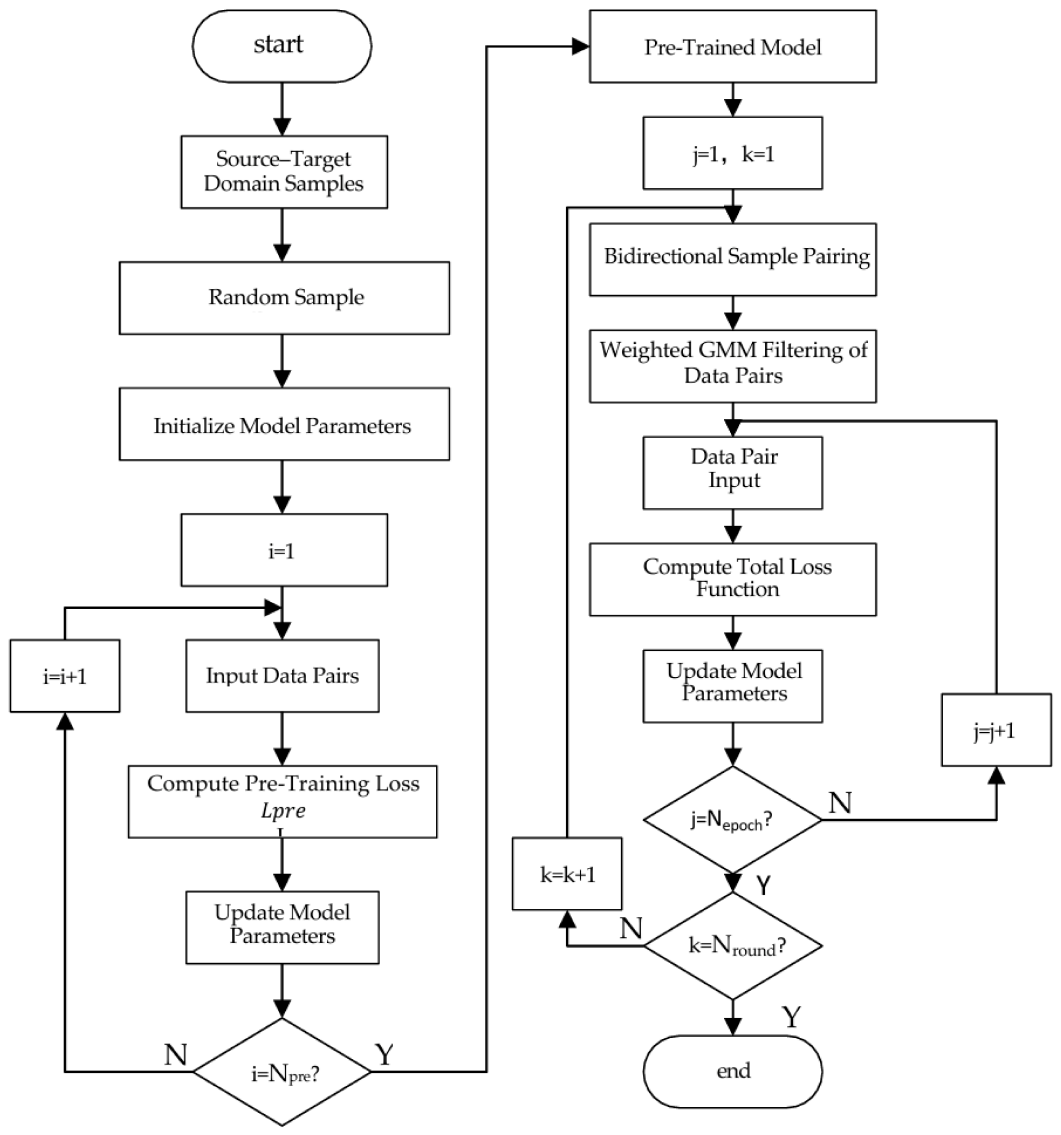
Cross-Condition Fault Diagnosis Workflow.

Figure 3 outlines the operational sequence. The superior performance of the proposed VICA-DA-ViT framework in cross-condition scenarios is fundamentally rooted in its mathematical optimization mechanisms. To provide a rigorous understanding of the framework’s inner workings, the following sections will detail the mathematical formulations of the GMM-based pseudo-label filtering, the cross-domain attention mechanism, and the dual-loss joint optimization strategy.

While Figure 3 provides a visual overview of the integrated diagnostic workflow, the specific execution logic and parameter updates are further detailed in the following operational stages. To ensure the reproducibility of the VICA-DA-ViT framework, the step-by-step implementation procedure—from initial data loading to the final output of the fault diagnosis model—is summarized in Table 2.

## 3. Experimental Results and Analysis

### 3.1. Experimental Setup

**Figure 4:**
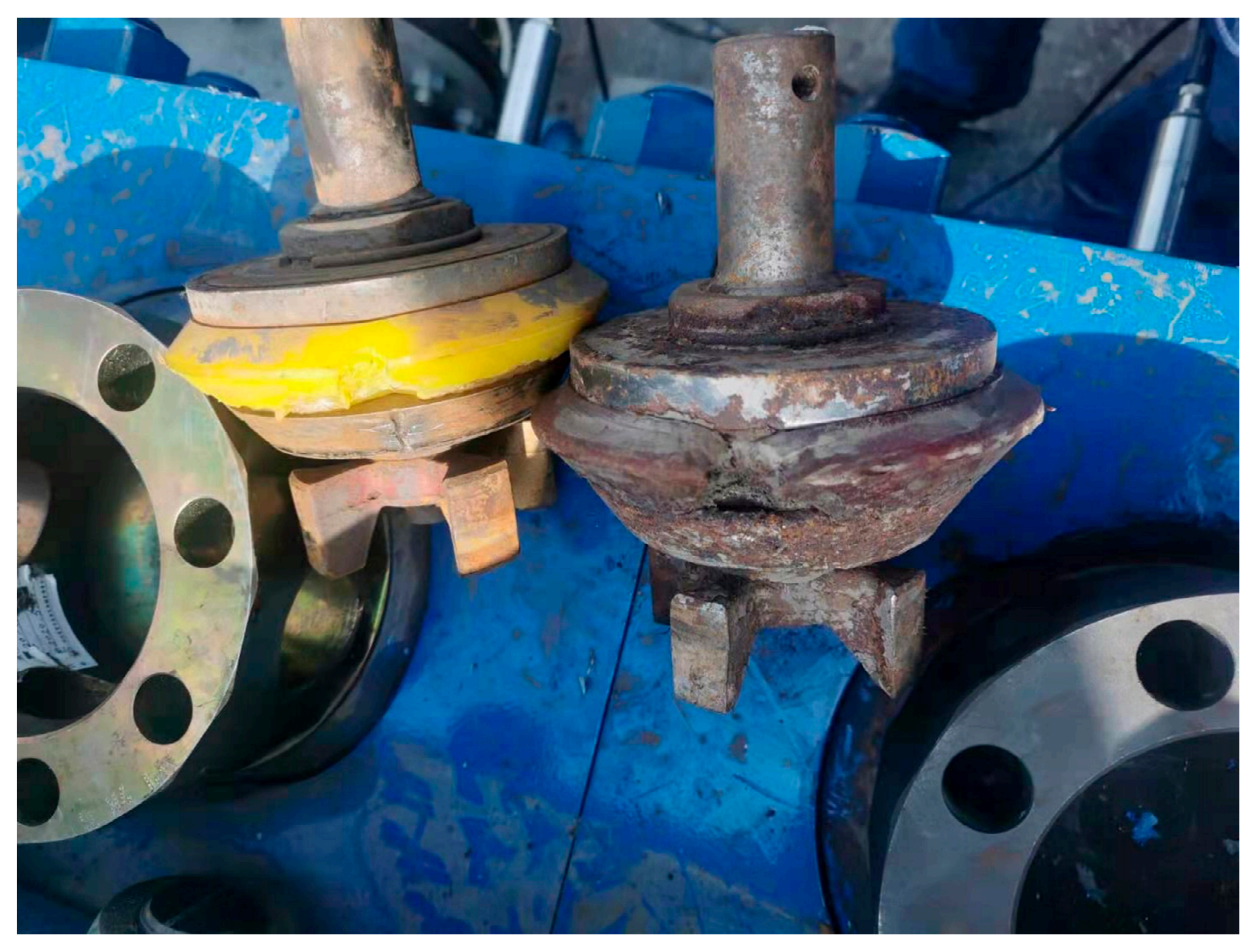
Photograph of a representative damaged valve component used in the fault simulation experiment.

The test object in this study is a HH2400 high-pressure quintuplex drilling pump (Sichuan Honghua Petroleum Equipment Co., Ltd., Guanghan, China). It comprises five independent fluid cylinders, each equipped with one inlet valve and one discharge valve, resulting in ten valves in total. In practical operation, inlet and discharge valves are prone to erosion, wear, and sealing failure under long-term high-pressure and high-flow-rate conditions. To simulate valve-related faults, artificially damaged inlet and/or discharge valve components were installed in Cylinders 1–3. A representative damaged valve component used in the fault simulation experiment is shown in Figure 4. Four health conditions were defined in this study: normal condition (N), inlet-valve fault (S), discharge-valve fault (R), and dual-valve fault (W).

**Figure 5:**
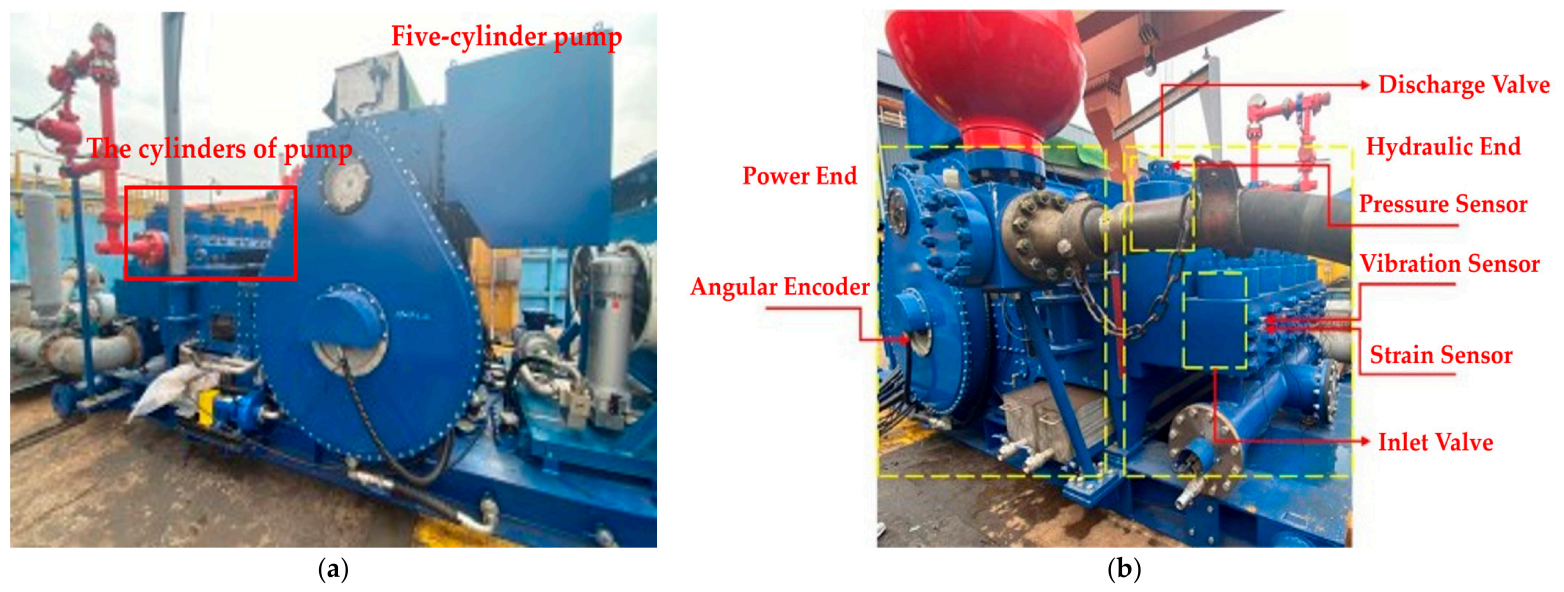
Site of the signal collection: (**a**) quintuplex pump test rig; (**b**) instrumented pump with sensor layout.

To acquire operating-condition information, a multi-sensor synchronous data acquisition system was deployed, with the sampling rate set to 1 kHz. Specifically, an angle encoder (E6CP-A, OMRON Corporation, Kyoto, Japan) is installed at the rotation center of the crankshaft to record the pump stroke cycles. Vibration sensors (S31D20, Yangzhou Xiyuan Electronic Technology Co., Ltd., Yangzhou, China) and strain sensors (CPR610-S01, Shenzhen Cooperation Sensors Instruments Co., Ltd., Shenzhen, China) are mounted on the outer surface of the inlet valve boxes to capture mechanical signatures, while pressure sensors (WIKA990.36, WIKA Alexander Wiegand SE & Co. KG, Klingenberg, Germany) are placed on the discharge valve boxes to monitor fluid pressure. The on-site acquisition setup is shown in Figure 5, and the detailed technical specifications of these sensors are summarized in Table 3.

Operating data were acquired under multiple stroke rates (50, 90, 110, and 130 SPM) and pump pressures (10, 20, 30, and 40 MPa). However, under higher-pressure conditions above 20 MPa, part of the vibration responses exceeded the measurement range of the vibration sensor, leading to saturated or clipped vibration waveforms and reduced feature reliability. Since the proposed diagnostic framework uses vibration signals as the primary input, these saturated high-pressure records were excluded from the final balanced diagnostic dataset. Therefore, the final experiments were conducted using the 90 and 110 SPM conditions under 10 and 20 MPa. Consequently, four operating conditions—90–10, 90–20, 110–10, and 110–20—were defined and denoted as Conditions (Domains) A–D, respectively. The specific parameters and labels for these four operating domains are summarized in Table 4.

Based on the available data acquired on-site from Cylinders 1–3, vibration signals are selected as the primary input for the diagnostic pipeline. This choice is grounded in the high sensitivity of vibration data to structural anomalies, providing the most distinct signatures for subsequent feature extraction and cross-condition fault diagnosis.

### 3.2. Data Preprocessing

**Figure 6:**
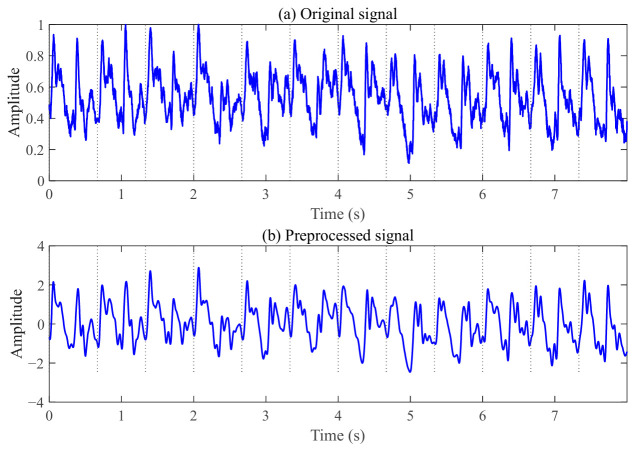
Time-domain waveforms of the vibration signal under 90 SPM and 10 MPa: (**a**) original signal; (**b**) preprocessed signal.

An 8000-data-point vibration segment is extracted from the vibration signal of Cylinder No. 1 under the 90 SPM and 10 MPa operating condition. Since the sampling frequency is 1 kHz, this segment corresponds to an 8 s time window. Figure 6a shows the time-domain waveform of the measured vibration signal. A five-level wavelet denoising procedure followed by mean removal is applied to the raw signal to suppress noise and satisfy the input requirements of subsequent VMD decomposition. The time-domain waveform of the preprocessed signal is shown in Figure 6b. In Figure 6, the horizontal axis is expressed in seconds, and the dotted vertical lines indicate crankshaft revolutions.

VMD is applied to the preprocessed vibration signal. To avoid insufficient decomposition or mode splitting, the mode number K is determined by inspecting the estimated center frequencies under different candidate K values. Specifically, K is adjusted based on whether adjacent center frequencies become excessively close, which indicates redundant or over-split modes. Table 5 lists the center frequencies of the obtained modes for different K values.

**Figure 7:**
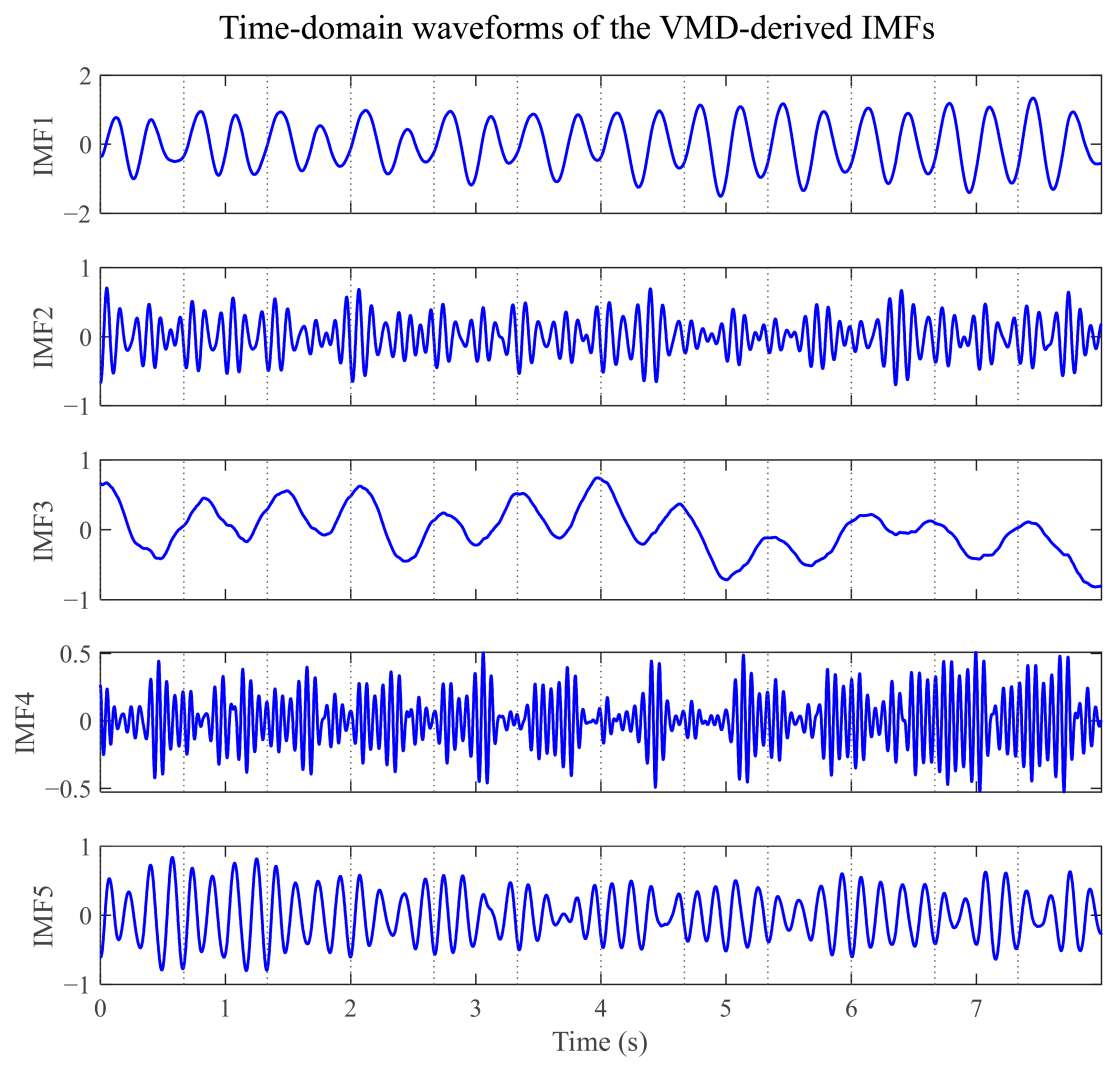
Time-domain waveforms of the IMFs obtained by VMD.

The selection of the VMD decomposition level K is critical for ensuring the integrity of fault feature extraction. According to the center frequency distribution in Table 5, when K is set to a low value (such as 2, 3, or 4), the model suffers from under-decomposition, where the insufficient mode separation causes distinct physical components—such as valve impact signatures and mechanical background noise—to be compressed into a single IMF, thereby masking critical diagnostic information. Conversely, when K = 6, the center frequencies of several modes become overly close (exhibiting a relative frequency gap insufficient to prevent spectral overlap), which triggers mode splitting and generates redundant components that fragment the signal energy. Consequently, K = 5 is identified as the optimal decomposition level because it balances frequency resolution and mode stability. The resulting time-domain waveforms of the five IMFs are shown in Figure 7, where the horizontal axis is expressed in seconds and the dotted vertical lines indicate crankshaft revolutions.

**Figure 8:**
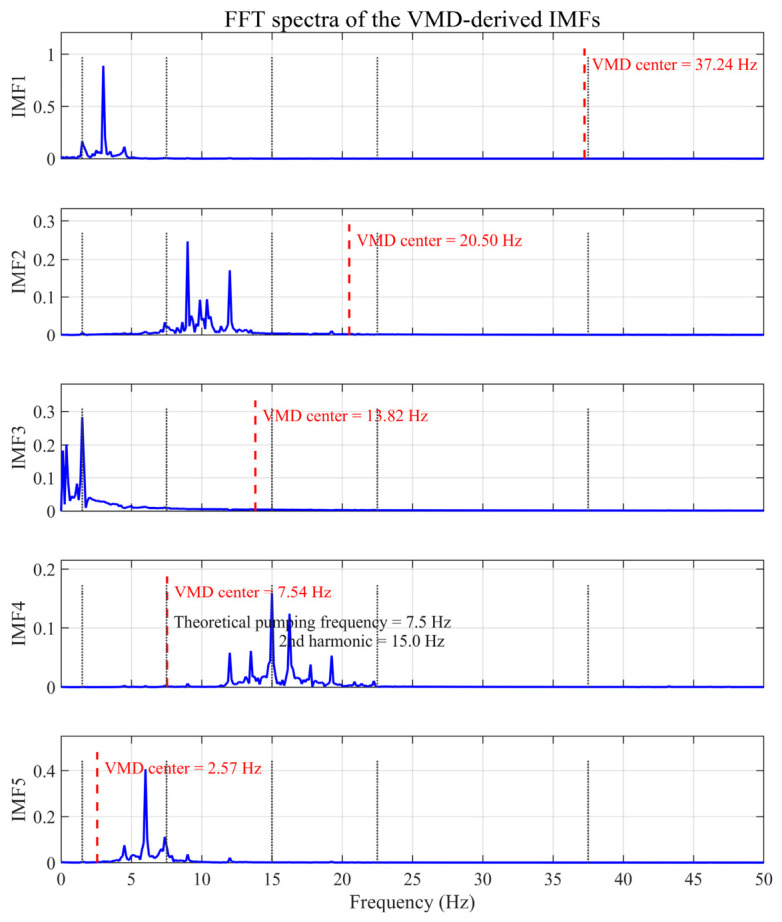
FFT spectra of the IMFs obtained by VMD under the 90 SPM and 10 MPa operating condition.

To further analyze the frequency characteristics of the VMD decomposition results, the FFT spectra of the IMFs are shown in Figure 8. At 90 SPM, the crankshaft rotating frequency is 1.5 Hz, and the main pumping frequency of the quintuplex pump is 7.5 Hz. The VMD center frequency of 7.54 Hz is close to the theoretical pumping frequency, while 37.24 Hz is close to its fifth harmonic. This indicates that the selected K = 5 can capture physically meaningful frequency components related to pump operation. It should be noted that the VMD center frequency represents an energy-weighted characteristic frequency and is not necessarily identical to the dominant FFT peak.

After the frequency-domain analysis, kurtosis is computed for each IMF to characterize its impulsiveness, and the Pearson correlation coefficient with the preprocessed signal is calculated to quantify signal relevance. By jointly considering kurtosis and correlation, informative IMFs can be selected for signal reconstruction while reducing the influence of irrelevant components. Table 6 summarizes the kurtosis and correlation values for each IMF.

**Figure 9:**
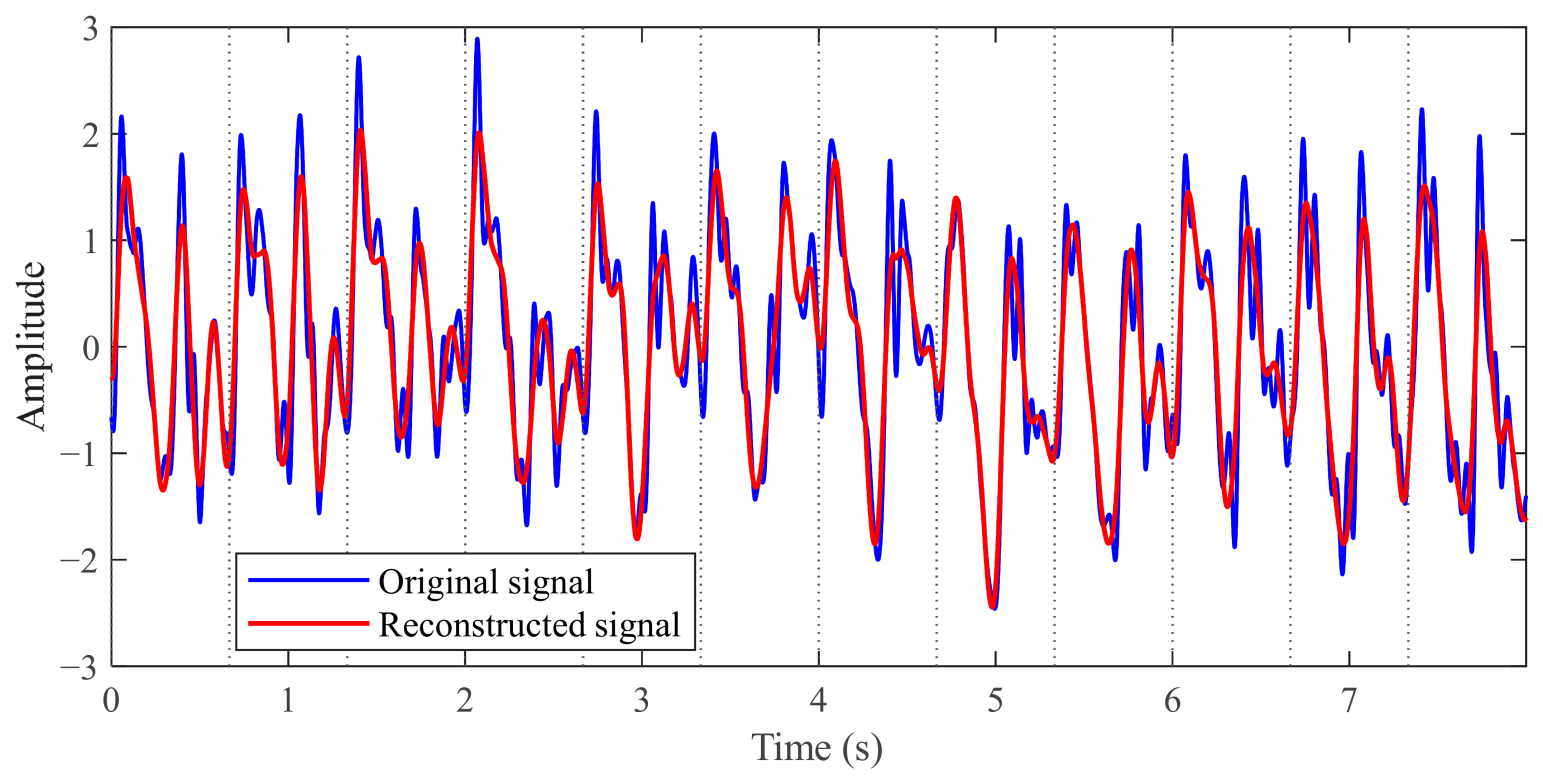
Comparison between the original signal and the reconstructed signal.

Based on the kurtosis and correlation analysis in Table 6, IMF1, IMF3, and IMF5 are retained for signal reconstruction, while the remaining components (IMF2 and IMF4) are combined to form a virtual noise channel. Figure 9 compares the original signal with the reconstructed signal.

**Figure 10:**
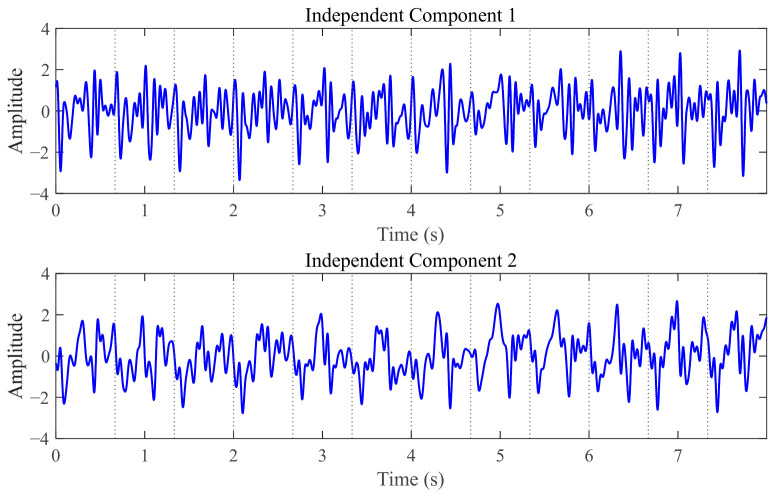
Two independent components separated by FastICA.

The reconstructed signal and the virtual noise channel are then fed into the FastICA algorithm for unmixing, yielding two separated independent components (ICs), as shown in Figure 10. For evaluation, the correlation coefficients between the separated components and the original signal, together with their kurtosis values, are reported in Table 7.

A larger absolute correlation coefficient indicates a stronger similarity between a separated component and the original composite signal, while kurtosis reflects the degree of impulsiveness (non-Gaussianity). As shown in Table 7, although IC2 exhibits a slightly higher kurtosis, IC1 possesses a significantly larger absolute correlation coefficient (0.98) compared to IC2 (0.18). This demonstrates that IC1 retains the clear majority of the source signal’s energy and captures the dominant impact-related vibration signatures of the target cylinder, whereas IC2 primarily isolates secondary crosstalk or noise. Consequently, IC1 is identified as the successfully separated target component and is selected for subsequent diagnostic analysis.

**Figure 11:**
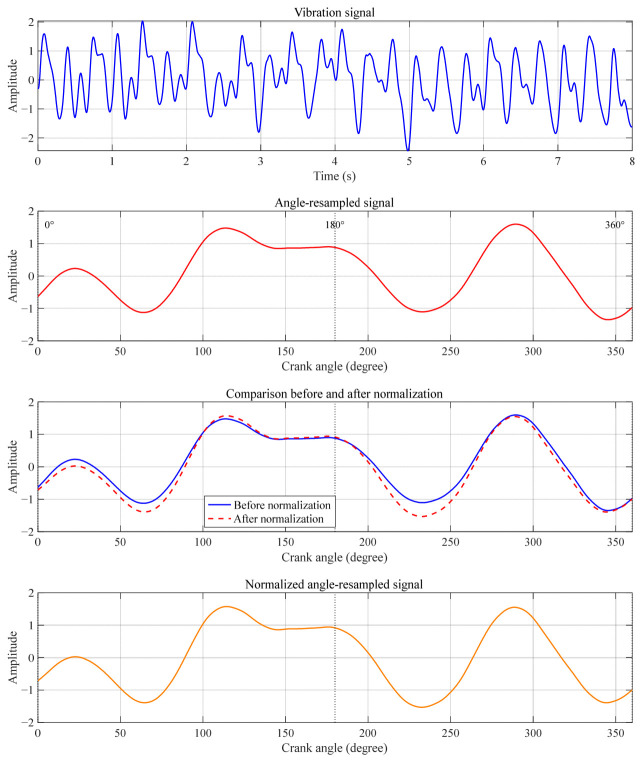
Illustration of angular-domain resampling and Z-score normalization (example at 90 SPM).

The crosstalk-suppressed vibration component (IC1) obtained by VMD–FastICA is then used as the input for angular-domain resampling and normalization. Figure 11 compares the one-cycle waveforms before and after angular-domain resampling (and subsequent normalization) under a 90 SPM operating condition.

Figure 11 shows that angular-domain resampling aligns the vibration signal to a fixed number of angular points per revolution, thereby reducing speed-induced variability and improving cycle-to-cycle comparability. In this study, each revolution is represented over 0–360° and resampled to 1800 angular points. Subsequently, Z-score normalization standardizes the signal amplitude by enforcing zero mean and unit variance. In this example, the normalized amplitude mainly falls within approximately −2 to 4.

### 3.3. Performance Analysis of the Proposed Cross-Domain ViT Model

#### 3.3.1. Input Construction and Preprocessing

**Figure 12:**
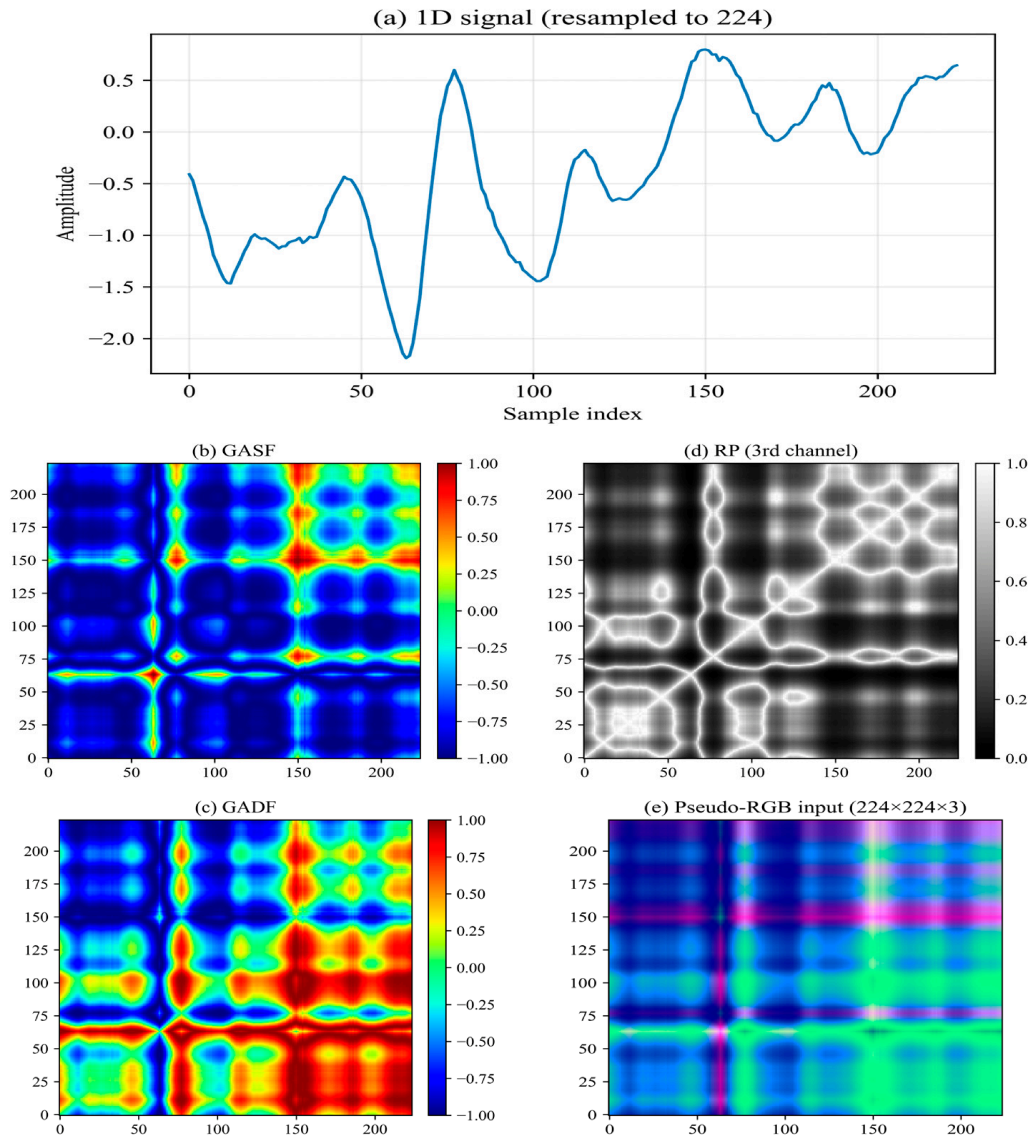
1D-to-2D transformation of the vibration signal for ViT input: (**a**) 1D signal (resampled to 224); (**b**) GASF; (**c**) GADF; (**d**) RP (3rd channel); (**e**) pseudo-RGB input (224 × 224 × 3).

To avoid ambiguity between angular-domain resampling and image input construction, the input construction procedure is clarified as follows. After VMD–FastICA-based crosstalk suppression, the vibration signal is segmented according to encoder pulses, and each complete crankshaft revolution is mapped onto a uniform angular grid of 1800 points for cycle alignment and phase normalization. This 1800-point angular-domain representation is used as an intermediate cycle-aligned representation rather than the final model input. For GAF–RP image construction, each cycle-aligned signal segment is standardized using Z-score normalization and uniformly downsampled to *n* = 224. The resulting one-dimensional sequence is then converted into three complementary two-dimensional maps, namely GASF, GADF, and RP, which are stacked to form a pseudo-RGB input of 224 × 224 × 3. The image is then fed into the ViT backbone with a patch size of P = 16, resulting in 14 × 14 image patches. Figure 12 shows an example of the 1D-to-2D conversion.

To evaluate the proposed ViT-based domain-adaptive method, the baseline performance of the pretrained model is assessed, then followed by cross-operating-condition experiments using limited labeled fault and normal data. The method is compared with representative cross-domain classification approaches, and cross-cylinder experiments are conducted to test generalization.

The diagnostic performance of the VICA-DA-ViT framework is evaluated using the four operating domains (A–D) and four health states (N, S, R, and W) defined in Section 3.1. These health states, referred to as Fault Types, represent different physical conditions: normal (N), inlet-valve fault (S), discharge-valve fault (R), and dual-valve fault (W). For each operating domain, 500 cycle-aligned signal segments are utilized, with a balanced distribution of 125 signal segments for each health condition. To avoid ambiguity, the term “signal segment” is used here to denote one training/testing instance, rather than an individual sampling point. For each domain, the signal segments are divided into training, validation, and test subsets at an approximate ratio of 4:1:1. Specifically, for each fault category, 83 signal segments are used for training, 21 for validation, and 21 for testing. Therefore, each operating domain contains 332 training segments, 84 validation segments, and 84 test segments. To reduce the risk of data leakage, the training, validation, and test subsets were kept strictly independent during model training and evaluation. No signal segment used for testing was included in the training or validation subsets. The specific sample counts for each subset across the four domains are summarized in Table 8.

For each operating domain and health condition, the original continuous vibration recordings were first divided into cycle-aligned signal segments according to the crankshaft revolution information provided by the angle encoder. The segments used in this study were constructed as non-overlapping cycle-aligned blocks. To prevent potential data leakage, the partitioning was performed at the continuous-recording/block level before model training. Therefore, signal segments originating from the same continuous recording block or the same crankshaft revolution were assigned to only one subset and could not appear simultaneously in the training, validation, and test subsets.

**Figure 13:**
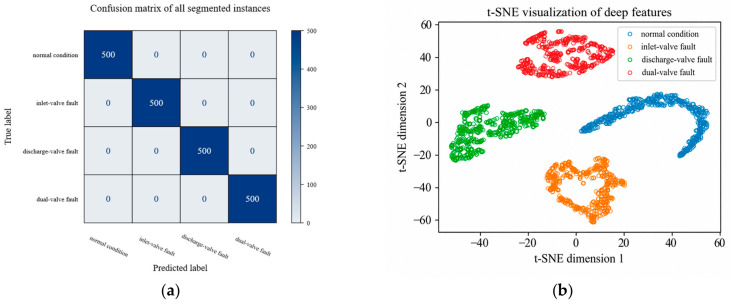
Feature-separability visualization generated from the complete set of 2000 segmented instances: (**a**) confusion matrix of all segmented instances; (**b**) t-SNE visualization.

After input construction and dataset partitioning, the processed signal segments are used for feature extraction and classification by the proposed VICA-DA-ViT framework. Figure 13 presents the confusion matrix and t-SNE visualization generated from the complete set of 2000 segmented instances across the four operating domains. The four health conditions include normal condition, inlet-valve fault, discharge-valve fault, and dual-valve fault. This figure is used to illustrate the separability of the extracted features after preprocessing and feature encoding, rather than to report the final independent test-set performance.

The t-SNE visualization was generated using deep features extracted from the penultimate layer of the trained model. The main t-SNE parameters were set as follows: perplexity = 30, learning rate = 200, maximum iterations = 1000, and random seed = 42.

Furthermore, the t-SNE visualization in Figure 13b provides an intuitive representation of the learned feature distribution. Deep features belonging to the same fault class form compact clusters, while different fault categories show clear spatial separation. This separability indicates that the combination of angular-domain resampling, GAF–RP feature encoding, and the VICA-DA-ViT framework can reduce speed-induced non-stationarity and enhance fault-discriminative feature representation. The final diagnostic performance is evaluated using the independent test subsets defined by the 4:1:1 partitioning protocol and is reported in Table 9, Table 10 and Table 11. In Table 9, Table 10, Table 11 and Table 12, the best, second-best, and third-best results in each column are highlighted in bold, italic, and underline, respectively.

All experiments were implemented in Python 3.10 using the PyTorch 2.10.0 as tbe deep learning framework. The proposed VICA-DA-ViT framework uses a standard ViT backbone for pseudo-RGB vibration-image feature extraction. Consistent with the GAF–RP input construction described above, the input image size is 224 × 224 × 3, and the patch size is set to 16 × 16, resulting in 14 × 14 image patches. The embedding dimension is 768, the number of Transformer encoder layers is 12, the number of attention heads is 12, the MLP ratio is 3, and the number of output classes is 4. The model is trained using the Adam optimizer with an initial learning rate of 1 × 10^−5^ and a batch size of 256. The number of domain-adversarial pretraining epochs is set to 100, the number of training epochs in each round is set to 30, and the number of iterative training rounds is set to 10. The label smoothing factor is set to 0.15. Dropout and Drop Path are both used for regularization, with the dropout ratio of the linear and attention modules set to 0.5 and the Drop Path ratio of the Transformer encoder set to 0.5. Runtime performance was measured on the same experimental platform equipped with an NVIDIA GeForce RTX 4060 Laptop GPU (NVIDIA Corporation, Santa Clara, CA, USA) with 8 GB of VRAM. For one cross-condition transfer task, the average training time was approximately 40 min, and the average single-segment inference time was within 10 ms.

#### 3.3.2. Pretrained Model Performance

To evaluate the initial cross-condition generalization of the pretrained models, data from Domain A (90 SPM–10 MPa) are used for training, and the trained models are directly tested on the other domains before applying the proposed cross-operating-condition diagnosis strategy. The performance comparison is reported in Table 9. 

As shown in Table 9, while most compared models achieve high accuracy on the source domain (A), their performance exhibits varying degrees of degradation when applied to cross-condition test domains, indicating a significant domain shift.

Notably, Multi-scale 1D CNN achieves the highest source-domain accuracy of 99.22%, followed by ResNet-18 (98.86%) and 1D CNN (98.44%). However, these models show limited robustness against condition variations. In contrast, although ViT is less competitive on the source domain than several CNN-based architectures, it yields the highest average accuracy (68.48%) across the four domains. This suggests that the ViT architecture demonstrates superior cross-condition generalization in this diagnostic task. The observed performance gap is consistent with the characteristic that Transformer-based models possess a higher capacity for capturing long-range dependencies, which potentially aids in identifying more invariant features despite the challenges of limited training data.

To further examine the cross-condition behavior of ViT under different source domains, ViT is pretrained on each single domain (A–D) and evaluated on the remaining domains. The results are summarized in Table 10.

As shown in Table 10, the cross-domain performance of ViT depends strongly on the selected source domain and exhibits clear asymmetry. Pretraining on Domain D achieves the highest average accuracy (71.10%), while Domain C yields the lowest (63.85%). In particular, transfer between certain domain pairs (e.g., D→B vs. B→D) shows noticeable discrepancies, indicating a non-negligible domain shift across operating conditions.

#### 3.3.3. Cross-Condition Experiment

In high-pressure quintuplex pumps operating under multiple conditions, variations in stroke rate and pressure can cause pronounced distribution shifts in vibration signals, making models trained under a single condition difficult to generalize to others. Therefore, cross-condition transfer learning is practically important for reliable fault diagnosis. In this experiment, the model is trained using labeled data from a known operating condition and then transferred to an unseen condition to evaluate its diagnostic capability under domain shift.

The experiment adopts the proposed ViT-based domain-adaptive framework, which integrates multi-head self-attention and cross-attention, together with bidirectional sample pairing and GMM-based filtering for cross-domain alignment and noise reduction. Comparative experiments are conducted against multiple representative cross-domain classification methods to validate the effectiveness of the proposed approach for cross-condition pump fault diagnosis. The results are summarized in Table 11.

Taking A→B as an example, the model is trained using only Domain A data (Cylinder 1, 90 SPM–10 MPa) and then evaluated on Domain B (110 SPM–10 MPa). This setting simulates a practical scenario where only historical vibration data under one operating condition are available, while the diagnostic model is required to correctly identify faults when the operating condition changes.

To further quantify the individual contribution of each component within the proposed framework—specifically the signal denoising module (VMD–FastICA), image encoding module (GAF), and domain adaptation modules (WBP and GMM-W)—a systematic ablation study was conducted under a pooled-domain evaluation protocol. Different from the cross-condition transfer evaluation in Table 11, where the “Average” denotes the mean diagnostic accuracy over different source–target transfer pairs, the “Acc” in Table 12 represents the classification accuracy of each ablation configuration on the pooled-domain test subset. Specifically, signal segments from Domains A, B, C, and D were combined and then divided into training, validation, and test subsets using the same 4:1:1 partitioning ratio. Therefore, Table 12 is used to evaluate the contribution of each module under the pooled-domain classification setting, while the final cross-condition transfer performance is reported in Table 11.

To quantitatively evaluate the diagnostic performance and the effectiveness of each component, four standard classification metrics are employed. These metrics are calculated based on the confusion matrix, comprising True Positives (TP), True Negatives (TN), False Positives (FP), and False Negatives (FN). The definitions are as follows:

Accuracy (Acc): The ratio of correctly predicted samples to the total number of samples.

(19) $$Acc=\frac{TP+TN}{TP+TN+FP+FN}\times 100\%$$

Precision (Prec): The ratio of correctly predicted positive observations to the total predicted positives.

(20) $$Prec=\frac{TP}{TP+FP}\times 100\%$$

Recall (Rec): The ratio of correctly predicted positive observations to all observations in the actual class.

(21) $$Rec=\frac{TP}{TP+FN}\times 100\%$$

F1-Score (F1): The harmonic mean of Precision and Recall, providing a balanced measure of the model’s performance.

(22) $$F1=\frac{2\times Prec\times Rec}{Prec+Rec}\times 100\%$$

Improvement in Accuracy ΔAcc: The absolute increase in accuracy compared to the baseline ViT model.

(23) $$\Delta Acc=Acc_{variant}-Acc_{baseline}$$

**Table 12:** Ablation study of key components under the pooled-domain evaluation protocol.

Configuration	Acc	Prec	Rec	F1	ΔAcc
ViT (baseline)	82.5	81.2	80.8	81.0	+0.0
ViT + Weighted bidirectional pairing (WBP)	85.1	84.3	84.0	84.1	+2.6
ViT + GMM-based confidence weighting (GMM-W)	86.4	85.8	85.5	85.6	+3.9
ViT + WBP + GMM-W	88.7	88.0	87.8	87.9	+6.2
GAF + ViT + WBP + GMM-W	90.2	89.5	89.3	89.4	+7.7
VMD–FastICA + ViT + WBP + GMM-W	*91.5*	*90.9*	*90.7*	*90.8*	*+9.0*
VMD–FastICA + GAF + ViT + WBP + GMM-W (Proposed)	**93.7**	**92.9**	**93.2**	**93.0**	**+11.2**

Following the overall comparisons in Table 11, Table 12 presents an ablation study to quantify the contribution of each component in the proposed framework. Starting from the ViT baseline, introducing the bidirectional pairing strategy and the GMM-based confidence weighting consistently improves the performance, and their combination yields a larger gain, indicating complementary effects. Incorporating the GAF-based 1D-to-2D representation further enhances cross-condition robustness, while VMD–FastICA preprocessing provides additional improvements by suppressing interference and highlighting fault-related components. Overall, under the pooled-domain evaluation protocol, the complete model achieves the best results across all metrics, demonstrating the contribution of jointly integrating preprocessing, representation construction, and domain-adaptation modules. It should be noted that these ablation results are used to analyze module effectiveness, whereas the final cross-condition transfer performance is reported separately in Table 11.

## 4. Conclusions

In this study, a comprehensive diagnostic framework, termed VICA-DA-ViT, was developed for high-pressure quintuplex pumps operating under complex conditions, covering the entire pipeline from signal preprocessing to intelligent fault identification. The VICA scheme, integrating VMD and FastICA, effectively suppresses inter-cylinder crosstalk and facilitates the precise extraction of cylinder-specific vibration components. By implementing angular-domain resampling (ADR) and normalization, the framework significantly mitigates speed-induced non-stationarity and enhances the consistency of cycle-wise signatures. The core diagnostic model, built upon a standard ViT backbone and enhanced with domain-adaptation strategies, successfully learns transferable, domain-invariant features across varying operations through domain-adversarial pretraining, bidirectional weighted pairing, and GMM-based confidence filtering. Experimental results demonstrate that the VICA-DA-ViT workflow achieves a high average cross-condition accuracy of 87.42%, providing a robust solution for fault diagnosis under noise, speed fluctuations, and limited labeled data. Ultimately, this study offers a highly practical and reliable methodology for the condition monitoring of multi-cylinder reciprocating machinery.

## Figures and Tables

**Table 1:** The detailed operational procedure of the proposed VMD–FastICA-based signal processing.

VMD–FastICA-Based Signal Processing
**Step 1.** Acquire the measured vibration signals and perform denoising and mean removal to reduce environmental and measurement noise, and satisfy the input requirements of VMD. **Step 2.** Decompose the preprocessed signal using VMD to obtain multiple IMFs modes and their corresponding center frequencies. **Step 3.** Adjust the mode number K by checking the separation of the estimated center frequencies: (a) if adjacent center frequencies become excessively close, reduce K; (b) otherwise, increase K and repeat the VMD decomposition. **Step 4.** Compute the kurtosis and Pearson correlation coefficient of each IMF to quantify its impulsiveness and similarity to the preprocessed signal. **Step 5.** Select informative IMFs according to kurtosis and correlation, and reconstruct them to form the observation signal. The remaining IMFs are combined to construct an auxiliary virtual channel. **Step 6.** Apply FastICA to the observation signal and the auxiliary channel to perform unmixing and obtain separated components with reduced crosstalk.

**Table 2:** The detailed execution procedure of the integrated VICA-DA-ViT framework.

VICA-DA-ViT-Based Fault Diagnosis Framework
**Step 1.** Load the preprocessed and standardized vibration dataset, and randomly select source-domain and target-domain samples to form initial data pairs. **Step 2.** Initialize the feature extractor, classifier, and domain discriminator. **Step 3.** Perform domain-adversarial pretraining for Npre iterations (or epochs) to obtain a pre-trained model. **Step 4.** Use the pretrained model to perform bidirectional pairing over all samples, and apply weighted GMM filtering to obtain a refined source–target pair set. **Step 5.** Feed the filtered data pairs into the ViT-based domain-adaptive diagnostic model, compute the total loss, and update model parameters for Nepoch epochs. **Step 6.** Update the target-domain pseudo-labels and confidence weights using the current model, and refresh the refined source–target pair set for the next round of training. **Step 7.** Repeat Steps 4–5 for Nround rounds. When the maximum round is reached, stop training and output the final fault diagnosis model.

**Table 3:** Technical specifications and installation positions of the sensors.

Sensor Type	Model	Installation Position	Measurement Range
Vibration Sensor	S31D20	Outer surface of inlet valve box	0–20 mm/s
Strain Sensor	CPR610-S01	Outer surface of inlet valve box	0–100 μθ
Pressure Sensor	WIKA990.36	Outer surface of discharge valve box	0–70 MPa
Angle Encoder	E6CP-A	Rotation center of the crankshaft	0–360°

**Table 4:** Detailed parameters of the four defined operating domains.

Domain	Stroke Rate (SPM)	Pump Pressure (MPa)	Combined Label
A	90	10	90–10
B	110	10	110–10
C	90	20	90–20
D	110	20	110–20

**Table 5:** Center frequencies of modal components for different K values.

K	Center Frequency/Hz						
2	13.49	3.03					
3	15.90	8.42	2.63				
4	21.75	13.99	7.53	2.58			
5	37.24	20.50	13.82	7.54	2.57		
6	82.27	36.06	19.80	13.26	6.89	2.54	
7	82.88	37.94	22.05	15.72	12.03	6.59	2.54

**Table 6:** Correlation coefficients and kurtosis values of the IMFs.

IMF	Correlation Coefficient	Kurtosis
1	0.78	1.87
2	0.37	2.63
3	0.50	2.61
4	0.25	2.90
5	0.44	2.16

**Table 7:** Correlation coefficients and kurtosis of FastICA-separated components.

Separated Signals	Correlation Coefficients	Kurtosis Values
IC1	−0.98	2.23
IC2	−0.18	2.52

**Table 8:** Cross-Operating-Condition Experimental Dataset.

Data Source	Operating Condition Domain	Sample Size	Fault Type	Sample Size
HH2400 quintuplex Pump	A: 90 SPM–10 MPa	500 samples per operating condition	Normal	125 samples per fault type under a single operating condition
	B: 110 SPM–10 MPa		Inlet-Valve Fault	
	C: 90 SPM–20 MPa		Discharge-Valve Fault	
	D: 110 SPM–20 MPa		Dual-Valve Fault	

**Table 9:** Performance Comparison of Pre-trained Models(%).

	Source Domain	A	B	C	D	Average
Method						
Swin Transformer [31]		97.44	63.45	50.09	52.04	*65.74*
PatchTST [32]		83.65	54.04	**62.86**	41.05	60.46
ConvNeXt [33]		94.49	52.45	57.23	45.21	62.37
InceptionTime [34]		98.20	*64.80*	45.47	44.81	63.33
1D CNN		98.44	**65.02**	48.20	47.82	64.99
Multi-scale 1D CNN [35]		**99.22**	58.28	50.88	*54.07*	65.50
ResNet-18 [36]		*98.86*	45.25	59.45	44.28	61.91
ViT		98.07	60.89	*60.25*	**54.49**	**68.48**

**Table 10:** Performance of ViT Pre-trained Models Under Different Source Domains(%).

	Target Domain	A	B	C	D	Average
Source Domain						
A		**98.07**	60.89	60.25	54.49	68.48
B		56.45	**98.22**	47.66	68.45	67.49
C		49.47	47.29	**98.83**	59.63	63.85
D		53.42	79.82	53.23	**98.23**	**71.10**

**Table 11:** Cross-Condition Diagnosis Accuracy (%) of Methods Under Different Source-Target Pairs.

	Cross-Condition Combination	A-B	A-C	A-D	B-C	B-D	C-D	Average
Method								
Source-Only ResNet		45.21	59.49	44.27	42.05	56.28	51.42	49.72
Source-Only ViT		57.83	60.23	53.40	47.62	64.45	43.65	54.55
DANN [37]		54.85	61.25	46.60	52.83	65.24	55.08	55.92
CDAN [38]		57.84	59.23	45.44	53.06	65.02	57.27	56.33
CDAN + E		57.46	60.22	47.09	53.83	65.28	56.27	56.65
DCAN [39]		68.87	74.23	53.20	62.22	63.82	61.28	63.94
DAGCN [40]		63.88	66.25	49.82	58.20	65.09	61.29	60.79
CD-Trans [41]		*87.68*	*72.68*	*77.25*	*65.05*	*78.89*	*71.40*	*75.48*
Proposed		**90.84**	**85.86**	**85.02**	**88.28**	**88.06**	**86.6**	**87.42**

## Data Availability

The data are not publicly available due to their containing information that could compromise the privacy of research participants.
